# PCE‐CfD and Long Covid: An NHS Service Evaluation on the Benefits of Using Person‐Centred Experiential Counselling for Depression With People With Long Covid

**DOI:** 10.1111/hex.70517

**Published:** 2025-12-13

**Authors:** Matthew R. Leavesley, Caroline Dugen‐Williams, David Dobel‐Ober, Alice Carson

**Affiliations:** ^1^ Shropshire, Telford & Wrekin NHS Talking Therapies Midlands Partnership NHS University Foundation Trust Shrewsbury UK; ^2^ School of Medicine, Faculty of Medicine & Health Sciences Keele University Keele UK; ^3^ Health and Social Sciences University of West of England Bristol UK; ^4^ Research and Innovation Department, St George's Hospital Midlands Partnership NHS University Foundation Trust Stafford UK

**Keywords:** depression, Long Covid, NHS Talking Therapies, person‐centred experiential counselling for depression, service evaluation

## Abstract

**Background:**

Long Covid is a condition affecting multiple organ systems and the mental health of patients. To address this, two National Health Service (NHS) services in the West Midlands developed an integrated long‐term conditions (LTCs) pathway, co‐produced between a ‘Post Covid’ service and an NHS Talking Therapy service for anxiety and depression (TTAD). Eligible people with Long Covid were offered person‐centred experiential counselling for depression (PCE‐CfD) to help improve their mental health. Despite limited evidence for PCE‐CfD in managing depression linked to LTCs, it was identified that a humanistic approach could help address the disrupted self‐narratives that existed with this cohort of patients.

**Objective:**

This NHS service evaluation investigated outcomes from clients with Long Covid who received PCE‐CfD, specifically the impact on reduced depression and anxiety symptoms, and improved social and occupational functioning. It analysed pre‐ and post‐treatment client/patient self‐reported data using routine outcome measures, including the PHQ‐9, GAD‐7 and WSAS.

**Methods:**

A non‐experimental cohort design was used to analyse anonymised routinely collected secondary data. Data that met the inclusion criteria were extracted for treatment delivered between August 2022 and October 2024. Paired *t*‐tests were used to examine whether there were significant improvements between pre‐ and post‐treatment outcome measures.

**Results:**

For people with Long Covid that completed treatment (*n* = 31), three *t*‐tests were completed showing a significant reduction from PCE‐CfD treatment, in depression (*p* < 0.01), anxiety (*p* < 0.01) and social functioning (*p* < 0.05). Cohen's *d* values indicated a very large effect size for a treatment effect in the reduction of depression (*d* = 2.1) and anxiety symptoms (*d* = 1.2). Recovery rates were analysed, using the NHS Talking Therapy recovery rate calculation, which showed that 83.87% of the people with Long Covid who were treated reached ‘recovery’, a much higher rate than the 48% NHS England target.

**Conclusion:**

This NHS service evaluation underscores the effectiveness of PCE‐CfD in reducing symptoms of depression and anxiety, as well as improving social and occupational functioning. These improvements observed in routine outcome measures highlight the benefits of offering a humanistic approach to people with Long Covid. These small but significant findings offer valuable insights into future service delivery and research in managing mental health challenges associated with chronic health conditions.

**Patient or Public Involvement and Engagement (PPIE) Contribution:**

This NHS service evaluation uses secondary data analysis, meaning PPIE did not take place in advance. However, PPIE discussions could arise when disseminating its findings that could lead to further research in this area.

## 1 Introduction

### 1.1 Background

On 11 March 2020, the World Health Organization (WHO) designated the outbreak of severe acute respiratory syndrome coronavirus 2 (SARS‐CoV‐2) a pandemic [1]. Commonly known as Covid‐19, the virus has caused 7 million deaths worldwide [2] and left many individuals suffering from persistent post‐viral symptoms [3]. These symptoms, lasting 12 weeks or more after the initial infection, can include fatigue, shortness of breath, cognitive impairment and muscle ache [4]. ‘Long Covid’, known formally as Post‐Acute Sequelae of SARS‐CoV‐2 infection [5], significantly diminishes quality of life [4, 6]. The Office for National Statistics (ONS) [7] estimates that there are 1.9 million people living with Long Covid in the United Kingdom, suggesting a 2.9% prevalence rate. Additionally, the long‐term physical effects of the condition negatively impact the mental health of people with Long Covid [8, 9]. Recently, studies have consistently shown people living with the condition have comorbidities, including anxiety, depression and post‐traumatic stress disorder (PTSD) [10, 11]. Patients who are experiencing recent‐onset depression or anxiety are likely to seek psychological support for their ongoing difficulties if their overall well‐being is being impacted [12]. However, this tendency to seek out support may be influenced by various psychosocial factors, including but not limited to gender, socio‐economic status and the availability of treatment.

### 1.2 Current Service Solutions and Treatment Controversies for Common Mental Health Problems

Evidence‐based clinical guidelines in the United Kingdom recommend psychological therapy as the primary treatment offer for common mental health problems [13, 14, 15]. The National Health Service (NHS) in England operates a locally delegated and established national service framework for the widespread delivery of NICE‐approved evidence‐based psychological therapies [16]. The framework and service model is called NHS Talking Therapies for anxiety and depression (NHS TTAD), known previously as Improving Access to Psychological Therapies (IAPT). It is widely commissioned across every Integrative Care Board (ICB—previously clinical commissioning groups) in England [17], with a more recent growth and delivery plan outlined within the NHS Long Term Plan in England [18], which included the expansion of psychological therapies for long‐term health conditions. NHS TTAD services provide evidence‐based psychological therapy for anxiety‐based problems, depression and PTSD. Therapies which are delivered are recommended by the National Institute for Health and Care Excellence (NICE) and the national competency frameworks [19, 20, 21]. For depression specifically, there is a choice of either Cognitive Behavioural Therapy (CBT), Individual Behavioural Activation, Interpersonal Therapy (IPT), Couples Therapy for Depression, Dynamic Interpersonal Therapy (DIT) or Person‐Centred Experiential Counselling for Depression (PCE‐CfD) [17]. Operationally, NHS TTAD services use an outcome‐based model, which has integrated data governance processes as well as robust clinical leadership requirements [17]. Services collect data at each therapy session by requesting clients to complete the 9‐item Patient Health Questionnaire (PHQ‐9) [22], focusing on depression and the 7‐item Generalised Anxiety Disorder questionnaire (GAD‐7) [23] for worry and generalised anxiety states. Services also request clients complete a questionnaire related to social and occupational functioning, called the Work and Social Adjustment Scale (WSAS) [24]. These are all self‐reports in nature and are completed and submitted by the client.

NHS TTAD services in FY 2024/2025 [25] were targeted at moving 48% of the population who accessed therapy towards clinically reliable recovery by the end of therapy and 67% towards a reliable improvement [17]. Recovery is achieved by measuring cohorts of clients who have accessed therapy and who were considered to be at case‐ness (above the agreed clinical cut‐off score on a psychological questionnaire) at the beginning of therapy and below case‐ness (below the cut‐off) at the end. To achieve recovery, a client/patient will need to have attended at least two therapy sessions and observed to move below the specific clinical cut‐off points of both a depression and anxiety questionnaire at the end of therapy [26]. Reliable improvement is measured through a nationally agreed reduction in scores on the questionnaires. The client's score does not have to move below the clinical cut‐off, but the score should be greater than the margin of error of the outcome measure [27]. Reliable recovery is indicated when the client has both scored below the clinical threshold (reached recovery) and shown reliable improvement in their clinical measures [17].

Controversially, the service model for NHS TTAD has come under extensive criticism for its neoliberal approach, using an outcome‐based, target‐driven model, with a focus on the delivery of CBT therapies [28, 29]. For example, measuring ‘recovery’ through self‐reported PHQ‐9 [22] and GAD‐7 [23] scores, without independent diagnostic validation, creates doubt as changes may result from natural improvement or regression to the mean, rather than treatment effects [29]. Furthermore, the positivist assumptions that underpin the orthodoxy of NHS TTAD services (to replicate the conditions of the underpinning randomised controlled trials [RCTs]) can lead to the exclusion of minoritised populations [30], due to biases that are generated from RCT methodologies. It is therefore important to the field of wider psychological practice to evaluate outlying populations, alongside the other modalities of therapies that are not necessarily represented in evidence‐based guidelines.

### 1.3 Aims and Objectives of the Service Evaluation

Unlike traditional research that aims to generate new knowledge, service evaluations assess existing service data to inform decision‐making and influence change [31]. Using data from routine outcome measures, patient feedback and/or staff input helps measure performance and recommend changes. Therefore, its main aim was to evaluate patient care, improve service delivery and support practice‐based evidence within the local NHS service. In this case, this service evaluation aimed to improve the future care offered to patients with long‐term conditions (LTCs), to learn lessons from the implementation of PCE‐CfD and to gauge whether PCE‐CfD may benefit those with Long Covid. The results could also influence future strategic planning. The epistemological assumptions were positivist in nature to follow the same philosophical stance adopted by the NHS Talking Therapy programme, due to being an evaluation of secondary data. The evaluation followed a quantitative non‐experimental naturalistic retrospective cohort design that was evaluative in nature.

## 2 Long Covid, Depression and PCE‐CfD

Depression‐based problems are reported as highly prevalent in people who experience Long Covid symptoms [32, 33]. People with long‐term health conditions (LTCs) experience lower recovery rates in the NHS TTAD programme [34, 35]. Evaluations of psychological therapies for depression, within NHS TTAD services, have observed no significant differences between counselling and CBT for depression, with both having similar treatment effects [36, 37]. There is also the so‐called ‘Dodo Bird effect’ [38], a tested hypothesis where all psychological therapies perform equally in the right conditions. Recent evaluations have also further supported this hypothesis, with more severe mental health problems, suggesting that it is the therapeutic relationship between patient and therapist that produces lasting change and psychological improvement [39]. Although improvements have been made in the investment towards relational therapies within the NHS Talking Therapies programme, counselling remains increasingly sidelined [40, 41], a trend recognised by the British Association for Counselling and Psychotherapy (BACP) [42]. Therefore, to ensure counselling didn't become superseded in the NHS and patient choice was maintained, the BACP developed a new evidence‐based manualised therapy for depression [43].

This unique therapy available in NHS TTAD services is called PCE‐CfD. Rooted philosophically within humanistic and positive psychology, PCE‐CfD is an amalgamation of person‐centred therapy [44] and emotion‐focused therapy [45]. Situated within a growth model of human experiencing [46], its main aim is to facilitate the uncovering and understanding of deeper emotions, allowing depressed patients to apply the insights they gain from therapy to transform their lives [47]. Moreover, the therapy addresses a patient's ‘self‐discrepancy’ [48], which is the gap between how a person sees themselves and how they think they should be. Targeting underlying emotional processes helps to reduce psychological distress and symptoms of depression [47]. However, as a therapy, it is distinguished from classical person‐centred practice [44] due to being time‐limited in nature (up to 20 sessions) and the need for collaborative goal setting between the patient and counsellor [43]. Furthermore, working within the biomedical model, that focuses on diagnosing and treating ‘symptoms’ based on standardised criteria, contradicts person‐centred theory [44] that emphasises a patient's subjective experience and growth potential [49]. Naturally, this struggle creates an ethical tension between the non‐directive, holistic ethos of person‐centred practice and working within an NHS TTAD service, that focuses on outcomes and ‘recovery’ [50]. However, such debates will continue to be had, with some in the profession offering a pragmatic stance on the issue [51] that sees PCE‐CfD as an effective bridge between valuing the subjective needs of patients on one‐hand and the need for evidence‐based practice on the other [40]. As a standalone therapy, PCE‐CfD is recommended within the standard NHS TTAD service framework [17], NICE guidelines for depression [15] and National Implementation Guidelines for LTCs [52]. However, due to the way in which the Covid‐19 pandemic rapidly took a grip of healthcare systems globally, novel approaches to treatment, including Long Covid, were required.

### 2.1 Supervision Within NHS TTAD Services

Effective supervision is crucial to the success of the NHS Talking Therapies programme, benefiting both patients and therapists [17]. Its primary purpose is to improve treatment outcomes, provide support to therapists and enable professional development. Milne's [53] definition of supervision outlines its three key functions as being: *Normative* that ensures adherence to ethical standards, professional guidelines and organisational policies, maintaining accountability and quality of care; *Restorative* that offers emotional support to therapists to help them manage the challenges and stresses of their work and *Formative* that focuses on developing therapists' skills, knowledge and competencies to enhance their professional growth and effectiveness. Particularly for counsellors, a key focus of supervision is to ensure adherence to the therapeutic model outlined in the PCE‐CfD Competence Framework. According to the BACP [54], practitioners are required to attend *a minimum* of 1.5 h of supervision per month. Furthermore, the NHS TTAD manual [17] recommends weekly supervision, consisting of at least 1 h of individual supervision with an experienced and trained NHS Talking Therapies supervisor. During the time of this evaluation, all PCE‐CfD therapists, including the Counselling Specialist, were in regular supervision.

### 2.2 The Development of a Long Covid Pathway

With increasing demand placed on the local NHS Primary and Secondary care services to meet the needs of people with Long Covid, a specialist service was commissioned. The Post‐Covid service, made up of a multidisciplinary team of professionals including Specialist Nurses, Occupational Therapists, Respiratory Physiotherapists, a GP and Clinical Psychologist, was established to holistically support the physical and mental health of its patients. In 2022, a Specialist Counselling role was included within existing funding to lead and manage an integrated LTC pathway with the local NHS TTAD service. This expansion meant people with Long Covid had a dedicated counselling resource that offered PCE‐CfD therapy to people with Long Covid, referred via the LTC pathway. During the implementation of the Post‐Covid service, the local NHS TTAD service was undergoing a service transformation and modernisation change programme. Although there were clinicians who were trained within the TTAD service on LTCs and modified therapies were being offered, there was no established integrated care pathway at the time. The absence of specific guidance on how to psychologically support patients with Long Covid and a paucity of literature meant an innovative approach was required. The rationale for choosing PCE‐CfD as the modality of choice to treat Long Covid was due to emerging evidence on how people living with chronic/persistent health conditions face not just a ‘broken’ body but also a disrupted self‐narrative causing depression [55], which in retrospect enhanced the rationale for a humanistic‐based psychological treatment.

## 3 Methods

### 3.1 Inclusion Criteria

This service evaluation reviewed cases following the delivery of therapy through secondary data analysis. All cases that were included in this service evaluation commenced their PCE‐CfD treatment after August 2022 and were delivered either via telephone, online or face‐to‐face. To be included within the evaluation, cases were required to satisfy the following inclusion criteria:

- The psychological therapy was focused on the symptoms of depression (with or without comorbid anxiety) that emerged as a result of a Long Covid diagnosis or self‐identification;
- The cases met the criteria for PCE‐CfD treatment, with the patient meeting the diagnostic criteria set out by NICE guidelines for depression [15] at Step 3. The minimal dataset is used as a tool to gauge at assessment the severity and persistence of depressive symptoms with the PHQ‐9 [22], needing to be 9 or above to qualify, as this indicates mild‐to‐moderate or moderate‐to‐severe depression;
- Cases had been discharged from the service following therapy; and
- The therapist who provided the therapy was either qualified in PCE‐CfD or in supervised training.

Any case with Long Covid that did not meet the inclusion criteria was removed from the dataset. The project was reviewed and supported by a Consultant Psychological Therapist and Clinical Lead (CDW), and monthly update reports were provided to ensure suitable governance processes were implemented and the evaluation met NHS standards.

### 3.2 Data Collection

Data collected consisted of patient self‐reported questionnaires that were completed before and after treatment. The dataset used was already submitted for public health surveillance purposes, under section 254 of the Health and Social Care Act (HSCA) 2012^1^. The minimum dataset (MDS) included outcome data from the Patient Health Questionnaire (PHQ‐9) [22], Generalised Anxiety Disorder scale (GAD‐7) [23] and WSAS [24]. A data technician (AC) was assigned to extract the data from the ‘backend’ of the electronic patient record system. They extracted key data, blind to the investigator, using a data extraction sheet and removed patient‐identifiable information. This was sent securely to the researcher (MRL), and the data was sent to be analysed by the data analyst (DDO). This service evaluation analysed secondary data of routine clinical outcome measures within NHS STW TTAD. Parameters set to extract the correct data were for people with Long Covid who commenced their PCE‐CfD treatment from 1 August 2022 until 31 October 2024. In total, 38 patients were identified who met the inclusion criteria (see 3.1 for more info) and were analysed. From those 38 patients, 7 were identified as not completing treatment and were discharged. These patients were excluded from all outcome analyses, but a comparison analysis was performed (see 4.5 for more info). In total, 9 PCE‐CfD therapists (qualified or in supervised training) provided therapy to people with Long Covid during the evaluation period.

### 3.3 Data Analysis

The main part of the data analysis consisted of comparing the scores of the three self‐reported questionnaires (PHQ‐9, GAD‐7 and WSAS) before and after treatment. Data were tested for normality: Shapiro–Wilk tests indicated that data were normally distributed in 4 of the 6 datasets; visual inspection of histograms and Q–Q plots suggested that data were close to a normal distribution in the other two datasets. Considering the central limit theorem and the sample sizes were > 30 (*n* = 31), the use of parametric tests was deemed appropriate and paired *t*‐tests were used. A Bonferroni correction was applied because of the increased risk of type I error associated with multiple tests [56]. Effect sizes were estimated using Cohen's *d*, with a *d* value near 0.2 considered a small effect, near 0.5 a medium effect, and near 0.8 a large effect [57]. The datasets for the non‐completers were not normally distributed, and because of the small sample size (*n* = 7), non‐parametric tests (Mann–Whitney U) [58] were used to compare score distributions between those who completed treatment and those who did not.

## 4 Results

### 4.1 Demographics of Clients, Origin of Referrals and NHS TTAD Outcome Measures

Table 1.

**Table 1:** Client data and recovery rates.

Client demographics		*N*	Minimum	Maximum	Median	Percentage
Age of client		31	23	69	48	
PCE‐CfD count		31	2	24	11	
Gender of clients	Female*	26				83.87%
	Male*	5				16.13%
Ethnicity of clients	White British or Irish	29				93.55%
	Asian or Asian British	1				3.23%
	Black or Black British	1				3.23%
Origin of referral	Secondary care single point of access	1				3.23%
	GP	1				3.23%
	Other	1				3.23%
	Self‐referral	18				58.06%
	Post‐Covid service	10				32.26%
	Total	31				100%
NHS TTAD outcome measures						
Recovered***	PHQ‐9					*82.14%*
	GAD‐7					*81.82%*
	Overall score**					*83.87%*
	NHS TTAD target (2019–2024)					*50%*
Reliable improvement****	PHQ‐9					*77.42%*
	GAD‐7					*70.97%*
	Overall score**					*90.32%*
	NHS TTAD target (2024–2025)					*67%*
Reliable recovery	PHQ‐9					*61.29%*
	GAD‐7					*54.84%*
	Overall score**					*77.42%*
	NHS TTAD target (2024–2025)					*48%*

*Note:* For the 9 PCE‐CfD therapists, the gender breakdown included 7 Females, 1 Male and 1 Non‐binary. The therapist ethnicity breakdown is 87.5% White British, Irish or European and 12.5% Asian or Asian British.

* Self‐identified, includes both cis‐gendered and transgender patients.

** Had a reliable recovery score on either the PHQ‐9 or the GAD‐7 [17].

*** Recovery percentages exclude any patients who were below the caseness threshold at the beginning, as per the Recovery calculation in the NHS TTAD Manual [17].

**** Reliable Recovery percentages include all patients, including those excluded from the Recovery percentage due to not starting at caseness.

### 4.2 Key Findings

The key findings from the analysis showed a significant reduction in symptoms for Long Covid clients following PCE‐CfD treatment. The routine outcome measures (PHQ‐9, GAD‐7 and WSAS) show strong clinical effectiveness (see Tables 1, 2, 3), with recovery rates of 83.87% overall. Reliable improvement was achieved by 90.32% of clients, exceeding the current 67% NHS TTAD target, while 77.42% met the criteria for reliable recovery, surpassing the 48% benchmark. These results indicate that PCE‐CfD therapy delivered strong outcomes across depression and anxiety metrics. In total, 31 clients who received PCE‐CfD therapy were included in the dataset. The clients had an average age of 48.6 years, with a majority being female (83.87%) and predominantly White British or Irish (93.55%). Most referrals came through self‐referral (58.06%) and the Post‐Covid service (32.26%). Finally, Long Covid clients had an average of 10.61 therapy sessions before they were discharged.

### 4.3 Standardised Measures (Table 2)

**Table 2:** PHQ‐9, GAD‐7 and WSAS pre‐ and post‐measures.

	Pre		Post		*t* (30)	*p* (two‐tailed)*	Cohen's *d*
	M	SD	M	SD			
PHQ‐9	17.48	4.891	7.90	3.259	11.795	< 0.001	2.119
GAD‐7	11.97	5.759	5.42	3.519	7.208	< 0.001	1.295
WSAS	22.61	9.670	15.61	9.429	3.410	0.002	0.62

* With Bonferroni correction, *α* = 0.0167. All three differences are significant.

**Figure 1:**
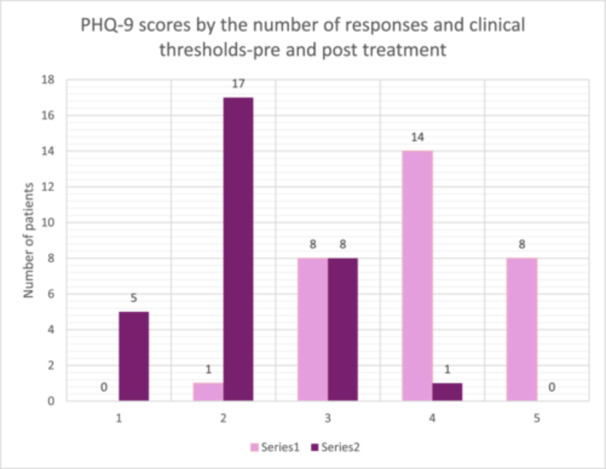
PHQ‐9 pre‐ and post‐measures by clinical thresholds.

**Figure 2:**
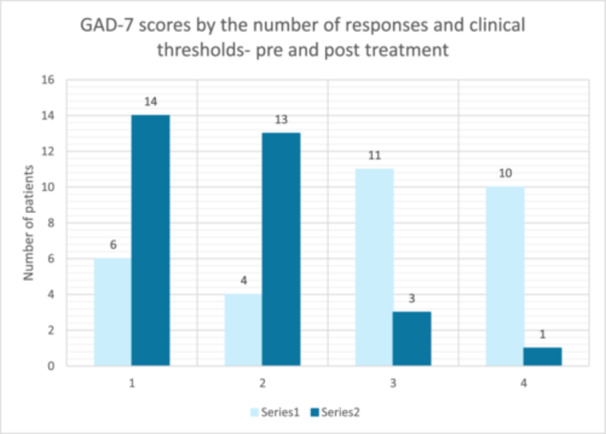
GAD‐7 pre‐ and post‐measures by clinical thresholds.

**Figure 3:**
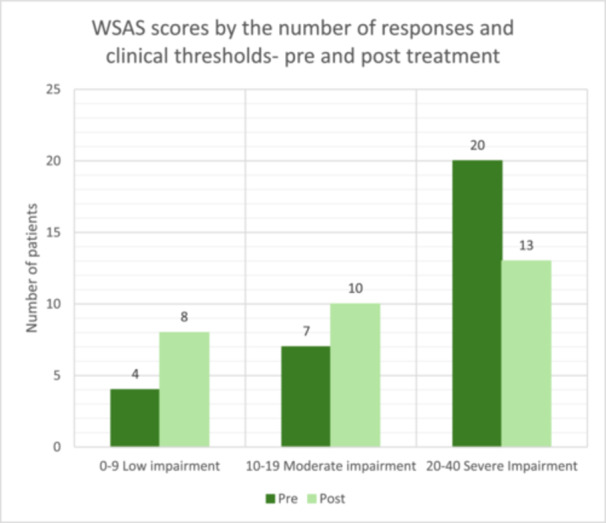
WSAS pre‐ and post‐measures by clinical thresholds.

Results from all three paired‐samples *t*‐tests, as shown in Figures 1, 2, 3, indicate that there was a significant reduction in symptoms following the PCE‐CfD intervention. Cohen's *d* [59] values indicate a large effect size for PHQ‐9 [22] and GAD‐7 [23] and a moderate effect for WSAS [24].

### 4.4 Clinical Thresholds

Figures 1, 2, 3.

### 4.5 Percentages of Patients Who Reached Recovery, Reliable Improvement and Reliable Recovery as Measured in NHS TTAD Services [17]

Table 3.

**Table 3:** Percentages of patients who recovered during treatment.

	Recovery*	Reliable improvement	Reliable recovery**
PHQ‐9	82.14%	77.42%	61.29%
GAD‐7	81.82%	70.97%	54.84%
Overall***	83.87%	90.32%	77.42%
*IAPT targets*		*67%*	*48%*

* Recovery percentages exclude any patients who were below the caseness threshold at the beginning, as per the Recovery calculation in the NHS TTAD Manual [17].

** Reliable Recovery percentages include all patients, including those excluded from the Recovery percentage due to not starting at caseness.

*** Had a reliable recovery score on either the PHQ‐9 or the GAD‐7 [17].

### 4.6 Comparison Between Patients Who Completed Treatment Versus Those Who Dropped Out

Overall, 31 patients completed treatment and 7 did not. Those who didn't complete treatment attended between 1 and 4 sessions, with the exception of one patient who attended 13 sessions, but their treatment was interrupted because their PCE‐CfD therapist went on long‐term sick leave (see Table 4).

**Table 4:** Age and number of CfD sessions attended by those who did not complete treatment.

	*N*	Minimum	Maximum	Median
Age of client	7	20	60	57
PCE‐CfD count	7	1	13	3

**Figure 4:**
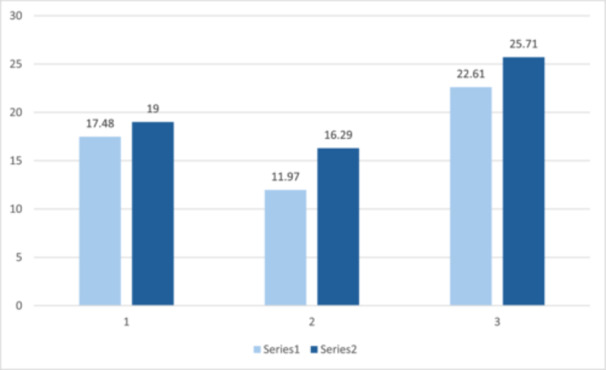
Mean scores on assessment for those who completed a course of treatment and those who did not.

As shown in Figure 4, those who went on to complete treatment had scored slightly lower at assessment than those who did not (for all three measures). Mann–Whitney U tests revealed no significant difference in score distributions between the two groups.

## 5 Discussion

### 5.1 Aims and Objectives of Service Evaluation

This service evaluation was conducted with the primary aim of enhancing patient care, improving service delivery and supporting evidence‐based practice within a local NHS TTAD service. Specifically, it sought to assess whether PCE‐CfD could benefit patients with a long‐term condition (LTC), specifically Long Covid. Through a non‐experimental retrospective cohort design, the evaluation aimed to learn lessons from current practice and determine the potential value of PCE‐CfD for this patient group.

### 5.2 Links to the Wider Literature

Despite the limitations of this evaluation, significant findings were observed with very large effect sizes on depression symptoms. The findings add to the growing theory of knowledge regarding the application of PCE‐CfD for Long Covid‐related depression; however, they would require replication and further research to be confident in treatment effects across different contexts. These findings are consistent with the wider literature on treatment effects of PCE‐CfD [36, 37]. Additionally, person‐centred experiential counselling is uniquely situated to address the delicate interplay between the physical and emotional symptoms of Long Covid. Burton et al.'s [9] study highlighted how the condition can trigger feelings of frustration, anxiety and grief, as patients struggle to adjust to reduced physical functioning and not knowing if they will recover. By focusing on the client's lived experience, PCE‐CfD validates these emotions, which can reduce feelings of isolation and promote psychological healing [43, 46]. Moreover, a study by Burton‐Fisher and Gordon [60] found that people with Long Covid felt ‘hopeful’ about recovery when receiving a combination of psychological and physiological treatments. However, evidence suggests that people with Long Covid face barriers when trying to access support for their condition, with some reporting that they receive no help at all [61].

With the transition into the post‐Covid‐19 period, emerging data suggest that the prevalence and recognition of Long Covid may be shifting due to changes in testing patterns, public awareness and diagnostic criteria [7]. Nonetheless, the psychological impact of enduring symptoms remain a concern, sustaining the need for therapies like PCE‐CfD, which are responsive to both emotional and physical health challenges that the findings from this service evaluation highlight.

The integrated pathway established between the NHS TTAD and Post‐Covid service in the West Midlands aimed to reduce these barriers. Patients who were identified at assessment with a mental health need were encouraged to self‐refer to the NHS TTAD service in the first instance. Thus, some of the people with Long Covid included in this evaluation were receiving multiple health treatments during their time in therapy. Consequently, studies show that interdisciplinary teamwork in managing chronic health conditions and targeted interventions improves patients' quality of life [62].

Additionally, research findings highlight how integrated services enhance patients' mental health outcomes and support better self‐management of their LTCs [63]. Regular follow‐ups, facilitation of referrals to specialists, and co‐ordination across health services help mitigate outcomes for patients with long‐term health conditions [64]. However, some LTCs are associated with greater intensity of care and poorer outcomes after therapy, leading to lower recovery rates within NHS TTAD services [34, 35]. While it is true that not all patients will require therapy to address their depressive symptoms, overall this service evaluation concurs with the body of evidence that highlights the potential benefits of service integration that focuses on the physical and mental health of patients with LTCs. Moreover, evidence highlighting that depression is frequently under‐recognised and insufficiently treated by non‐mental healthcare professionals [65] highlights the need to reduce barriers for patients. Achieving better detection rates and managing patient depression linked to their LTC (including Long Covid) requires a service redesign to be actioned [66]. This evaluation supports this notion, given how services can collaborate to ensure people with Long Covid with symptoms of depression are identified and discussed at regular multidisciplinary meetings. This approach aligns with current Long Covid research that argues for better collaboration and communication across healthcare professionals and settings [61].

When it comes to treating depression, the number of sessions offered varies according to NICE guidelines [15] and the NHS TTAD manual [17]. For mild‐to‐moderate depression at High Intensity level (Step 3), a treatment course of PCE‐CfD is typically up to 8 regular sessions [15]. However, for patients with comorbid mental and/or physical health problems, up to 20 sessions can be offered within an NHS TTAD service, depending on the problem descriptor and severity [17]. The 31 Long Covid cases featured in this evaluation, who completed treatment, averaged *10.61 sessions*. However, the challenges and barriers that LTC patients face when attending appointments emphasise the significance of providing adaptable, tailored therapy for individuals with LTCs alongside ensuring that staff and services are equipped with sufficient training and resources [67]. These findings correspond with existing research, although these were mainly focused on implementing low‐intensity interventions or CBT‐based treatments [63]. Our findings offer evidence that PCE‐CfD delivered at High Intensity (Step 3) could also be adapted for effective treatment of patients with comorbid LTCs, particularly Long Covid.

A consideration that must be made when selecting therapy for use within an NHS TTAD service is the requirement to meet national targets, including those relating to the efficacy of treatment. 48% of clients that access these services are targeted to reach reliable recovery, on at least one of the required clinical measures by the end of therapy and 67% are targeted to have reached reliable improvement on at least one of the measures [25]. The targets for NHS TTAD services historically have been that at least 50% of clients reach recovery [68], and these were updated to 48% when people had already received counselling interventions, so some of the data reported the 50% metric and some the 48% metric. Overall, the results were statistically above both values. Previous studies into the performance of NHS TTAD services when treating clients with LTCs have shown that patients with LTCs are less likely to reach these targets [34, 35]. Whilst our data cannot speak to all LTCs, they do indicate PCE‐CfD was an effective treatment option for clients with Long Covid in our pilot service. 83.87% of clients treated reached recovery, which is higher than the historical 50% target. Relative to the current targets, 90.32% of clients achieved reliable improvement and 77.42% reached reliable recovery on at least one of the measures, far higher than the targets of 67% and 48%, respectively. This data is not entirely comparable to recovery scores as published by NHS TTAD services, as we excluded clients who did not complete the treatment, as indicated by the therapist at discharge. However, national implementation guidelines count any clients who have had more than two treatment appointments and so would include clients who dropped out midway through treatment [17]. Despite this limitation, PCE‐CfD was deemed to be highly efficacious with people with Long Covid in this evaluation. Furthermore, our findings show that PCE‐CfD can be implemented within an NHS TTAD service without having a negative impact on the outcome measures, although our results suggest this requires an integrated LTC care pathway with wider interdisciplinary colleagues. Further experimental research is required to further these findings, with an intention‐to‐treat methodology, so all cases are included in the analysis.

Finally, considerations of the implementation of PCE‐CfD within LTCs' pathways may incur additional costs. Workforce retraining, supervision and adapting service models to a protocol‐driven CBT approach may prove challenging. Practical considerations need to ensure clinical efficacy across diverse LTC populations and assess whether PCE‐CfD can be integrated into existing NHS Talking Therapies frameworks without compromising service accessibility or treatment fidelity.

### 5.3 Strengths

We would argue that practice‐based observational evaluations, such as the one described here, can contribute to wider knowledge and theory development. Observational evaluations help democratise research by empowering individual clinicians to conduct meaningful studies, bypassing the complexities of large‐scale research studies that are often inaccessible to most practitioners. Democratising research could be part of a wider research strategy where highly accessible methods, such as observational studies, can feed into large trials and form a body of knowledge. This NHS service evaluation may help improve healthcare delivery and outcomes for people with Long Covid whose mental health has been impacted by their chronic health condition. It will help inform best practice for the local NHS TTAD moving forwards and future strategic direction, in understanding the role of PCE‐CfD within an LTC clinical pathway. Additionally, the data identifies key areas that can support better clinical decision‐making, more efficient use of LTC resources and the benefits of multidisciplinary working.

### 5.4 Limitations

Results should be interpreted with caution: this non‐experimental cohort study was based on a small sample in one geographical location of England, within the unique circumstances of an integrated LTC pathway being set up between a Post‐Covid service and an NHS TTAD service, and it was not suitably powered to draw wider inferences.

Furthermore, the study used an uncontrolled before‐and‐after design; this approach presents several limitations [69]. This study design is susceptible to overestimating treatment impact, as it does not account for changes occurring naturally over time, nor does it deal with regression to the mean. Without a control group, it is not possible to rule out the influence of external factors and establish a causal relationship between intervention and observed outcomes.

Confounding variables likely include prior learning, gender, ethnicity, attachment, readiness to change, current relational context, diversity, and therapists' personal impact. Unlike medication, therapists interact with and are shaped by the social world, with Shean [70] suggesting that therapist factors can account for 15%–30% of the variance in clinical outcomes. Close supervision, evaluating therapist allegiance to the therapy being evaluated, and monitoring therapist enthusiasm are some of the methods that can be employed to reduce performance bias.

It has been established [71] that bias can exist within psychotherapy studies, and the correlational effects of the intervention can be exaggerated in some cases. This is not limited only to observational cohort designs, and randomised trials experience the same inflated results when blinding is not applied [72].

Finally, as the project included all people accessing the service studied, any access bias—including through the referral process—was reflected in the study sample. Patients from ethnic minority backgrounds were under‐represented, with 93.55% of the sample being from White British or Irish backgrounds, most of them being female (84%).

## 6 Conclusions and Implications for Future Research

The findings of this NHS service evaluation have important implications for the future direction of potential research in this area, particularly in exploring the role of humanistic approaches like PCE‐CfD and in managing mental health challenges associated with chronic conditions, including Long Covid. Future research could expand on these findings by examining larger and more diverse patient cohorts to validate outcomes for a larger population. Additionally, longitudinal research could investigate the sustainability of the observed improvements over time, providing insight into the long‐term benefits of integrating such therapies into routine NHS care and LTC pathways. Comparative studies evaluating PCE‐CfD alongside other therapeutic approaches could also help refine best practices for supporting people with Long Covid. These directions would not only build upon the evidence base but also inform policies for further enhancement of NHS integrated LTC pathways between physical and mental health services.

This NHS service evaluation observed PCE‐CfD significantly reducing psychological distress and improving social and occupational functioning. The findings, although not generalisable, are encouraging and further research is recommended in this area. The significant improvements observed in patient‐reported outcomes provide valuable evidence supporting the continuation of PCE‐CfD being offered within NHS TTAD services for individuals experiencing mental health challenges related to chronic health conditions. The findings also highlight the potential of a humanistic approach in addressing disrupted self‐narratives and fostering recovery among this patient group. Future research could build upon these results by investigating larger cohorts, exploring longer‐term outcomes from follow‐up and comparing PCE‐CfD to alternative therapeutic modalities.

Responding to the needs of patients, using pragmatic approaches can respond to diverse groups in society and ensure the needs of people in marginalised groups are met [73]. We believe that no single therapy, in its purest form, can fully address the diverse needs of patients. Instead, a more pluralistic approach offers an integrative framework for generating knowledge and applying therapeutic interventions effectively. This method can help address some of the gender and ethnicity differences observed in this evaluation.

NB: The funding for the Long Covid Counselling Specialist role ended in 2024. However, at the time of writing, the integrated LTC pathway between the NHS TTAD and Post‐Covid service is still in place.

## Author Contributions

**Matthew R. Leavesley:** conceptualisation, investigation, project administration, writing – original draft, writing – review and editing. **Caroline Dugen‐Williams:** supervision, validation, writing – original draft, writing – review and editing draft. **David Dobel‐Ober:** formal analysis, validation, writing – original draft. **Alice Carson:** data curation, writing – original draft, writing – review and editing.

## Ethics Statement

The article describes a service evaluation, which received NHS Trust approval [*Project ref no: E2024/34*] in June 2024. The Health Research Authority (HRA) [74] decision‐making tool was used to guide decision‐making, with the project being classified as a service evaluation, and thus did not require formal ethical approval. This is due to the evaluation utilising existing data for healthcare purposes. Patients could opt out of their data being used for evaluative research purposes, as part of the NHS national data opt‐out scheme (NHS England National Data Opt‐Out [2024]. https://digital.nhs.uk/services/national‐data‐opt‐out#:~:text=The%20National%20Data%20Opt%2DOut,used%20for%20research%20and%20planning.&text=Patients%20can%20find%20out%20more,choice%20on%20the%20NHS%20website). Data included anonymised secondary data of completed cases, who were seen for psychological therapy within a commissioned Post‐Covid service, which was established following the NHS response to the impact of Covid‐19. Professional codes of practice (BABCP, BACP and BPS) were followed when undertaking this service evaluation.

## Consent

The Health Research Authority (HRA) [74] decision‐making tool was used to guide decision‐making, with the project being classified as a service evaluation, and thus did not require formal ethical approval. This is due to the evaluation utilising existing data for healthcare purposes. Patients could opt out of their data being used for evaluative research purposes, as part of the NHS national data opt‐out scheme (NHS England National Data Opt‐Out [2024]. https://digital.nhs.uk/services/national‐data‐opt‐out#:~:text=The%20National%20Data%20Opt%2DOut,used%20for%20research%20and%20planning.&text=Patients%20can%20find%20out%20more,choice%20on%20the%20NHS%20website).

## Conflicts of Interest

The authors declare no conflicts of interest.

## Data Availability

The data that support the findings of this study are available on request from the corresponding author. The data are not publicly available due to privacy or ethical restrictions.

## Endnotes

1 Under the General Data Protection Regulations (GDPR), the basis for processing these data was in accordance with Article 6 (1) (c), which relates to processing data necessary to comply with a legal obligation. NHS's lawful basis for processing special category data is GDPR Article 9 (2) (h) and Schedule 1, Part 1 (2) (2) (f) of the Data Protection Act 2018.
